# Osteoporotic Vertebral Compression Fracture and Single Balloon Extrapedicular Kyphoplasty: Findings and Technical Considerations

**DOI:** 10.29252/beat-080106

**Published:** 2020-01

**Authors:** Pankaj Kumar Mishra, Rishi Dwivedi, Charanjit Singh Dhillon

**Affiliations:** 1 *Department of Orthopedics, Gandhi Medical College and Hamidia Hospital Bhopal M.P., India*; 2 *Department of Spine Center, MIOT International Chennai, India*

**Keywords:** Uni-extrapedicular, Balloon kyphoplasty, Percutaneous, Compression fracture

## Abstract

**Objective:**

To evaluate the functional and radiological outcome of balloon kyphoplasty and to endorse the unilateral single balloon extrapedicular kyphoplasty as practically more feasible and safer method in comparison to the conventional methods.

**Methods:**

Totally, 81 patients were presented to our center with osteoporotic vertebral compression fracture. Among these, 59 patients (61 vertebrae) were enrolled with stable wedge osteoporotic compression fracture. Pre-operatively percentage of vertebral height loss and kyphotic angle were calculated and single balloon extrapedicular kyphoplasty was performed in all cases.

**Results:**

Postoperatively, anterior vertebral height improved to 79.61% of normal subjects. In our study, the mean segmental kyphosis correction following balloon kyphoplasty was 14.27°. Overall incidence of cement leak in our study was 15.25%.

**Conclusion:**

Although we encountered the few difficulties, but this technique holds the safety and feasibility measures. Furthermore, it is effective in restoring anterior vertebral height, alignment and angle of kyphosis.

## Introduction

Percutaneous cement augmentation is now an established mode of treatment of acute onset intractable pain due to osteoporotic vertebral fractures or chronic pain as a result of insufficient healing due to altered biomechanics or pseudoarthrosis [1,2]. Balloon kyphoplasty has superiority over vertebroplasty in improving the anterior vertebral height (sagittal balance) and less cement leakage [3, 4]. Balloon kyphoplasty involves inflation of balloon in fractured vertebra to elevate vertebral end plate [5, 6]. 

Symmetric and effective elevation of end plate needs two inflatable balloons to be placed through both the pedicle (transpedicular balloon kyphoplasty). Complications of balloon kyphoplasty range from mild increase in pain after procedure to cement leakage and pulmonary embolism. Cement leakage into paravertebral soft tissue occurs up to 51% in patients with no clinical significance. Paravertebral veins can lead to pulmonary embolism [7- 9]. Epidural space cement leakage can have serious outcomes like paraplegia. Chiras *et al*. reported 0.4% incidence of paraplegia as a result of epidural cement leakage [10]. This leak can occur as a result of breach in medial pedicle wall or needle traversing through lamina in patients with very narrow pedicle. 

Brugieres *et. al*. were the researchers who used the extrapedicular (transcostovertebral) approach first time for percutaneous biopsy from the central part of upper thoracic vertebrae [11]. In the subsequent years, this approach was utilized for the single balloon kyphoplasty via extrapedicular approach. Although the results of single balloon extrapedicular kyphoplasty is similar to two balloon transpedicular kyphoplasty, but it has the few advantages like short surgical time and less economic burden [12]. 

Standardization for single balloon extrapedicular kyphoplasty approach has not been made till now, due to variable trajectory (skin entry to centre of vertebra) at each level. So probably it could have been the reason that this approach is less practiced and need experienced hand, in comparison to two balloon transpedicular kyphoplasty [13]. This study was conducted to evaluate the functional and radiological outcome of balloon kyphoplasty and to endorse the unilateral extrapedicular kyphoplasty as practically more feasible and safer method compared to the conventional methods.

## Material and Methods

A total of 81 patients were presented to our centre with osteoporotic vertebral compression fracture from February 2016 to January 2018. Among these, 59 patients (61 vertebrae) who fullfilled our inclusion criteria underwent unilateral extrapedicular balloon kyphoplasty. Inclusion criteria were single level stable wedge osteoporotic compression fractures (not improved after conservative management) with intact posterior wall of lower thoraco-lumbar vertebrae, normal neurological status and without signs of infection. 

Patients of T9 or higher level of vertibra, history of coagulation disorders, pathological fracture and allergy to polymethylmethaacrylate, were excluded from the study. While 5 patients with involvement above D10, 2 patients with >2 vertebra involvements, 4 patients with posterior cortical breach and 3 patients who did not come for routine follow-up, were excluded from the study. One patient was found unfit for surgical intervention and 7 patients were not willing for any surgical procedure. 

**Fig. 1:**
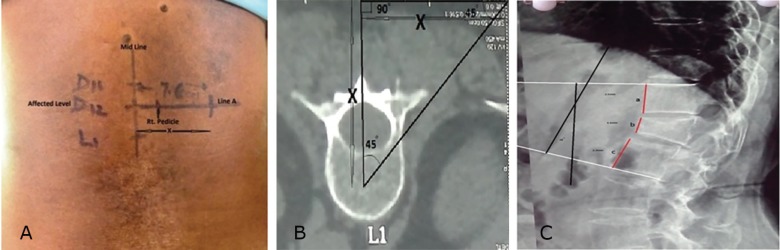
**A.** Determination of starting point for procedure surface marking, affected, proximal and distal levels were marked, a line A was drawn perpendicular to midline bisecting the affected level as seen on C-arm. Starting point was located at a distance of X cm on line A, where X=distance of center of affected vertebral body from dorsal skin surface (measured preoperatively on axial CT images. (c) Figure showing measurement of segmental kyphosis and percentage anterior vertebral height. Percentage anterior vertebral ht. at affected level=2xb/(a+c)x100. **B.** Schematic representation; axial CT section of L1 vertebra showing that midpoint of the vertebra to be reached by inserting needle at 45°, if starting point was chosen as described before. **C.** Figure showing measurement of segmental kyphosis and percentage anterior vertebral height. Percentage anterior vertebral ht. at affected level=2xb/(a+c)x100

All patients underwent complete clinical, laboratory and radiological evaluation to rule out other causes of vertebral fractures like multiple myeloma, and metastasis. X-ray and CT were done for all patients to determine involved vertebral level. Pre-operatively, percentage of vertebral height loss and kyphotic angle were calculated (Figure 1A). Percentage height loss was calculated by comparing the anterior vertebral height of involved level with mean of anterior vertebral height of proximal and distal un-involved levels. 

On Axial CT sections, distance of center of involved vertebra from skin was measured. Pain and quality of life were assessed by Visual Analogue Scale (VAS). All patients were explained in details about nature of ailment and available non-operative and operative treatment options. Detailed informed consent was taken for each case. Regarding the operative technique, under general anesthesia, the patients in prone position on a radiolucent table; C-arm was positioned in such a way that anteroposterior (AP) and lateral images were possible during the procedure and AP and lateral images were taken. 

Since we were apprehensive for the epidural leakage in this technique, we preferred the general anesthesia, so that in any emergency intervention could be implemented. Following disinfection and draping, mid-line was marked after obtaining a true AP fluoroscopy image of the affected level and later, a K-wire was aligned over the skin so that it appears to bisect the affected level. A horizontal line (Line A) was drawn along the skin using this wire (Figure 1B). 

**Fig. 2:**
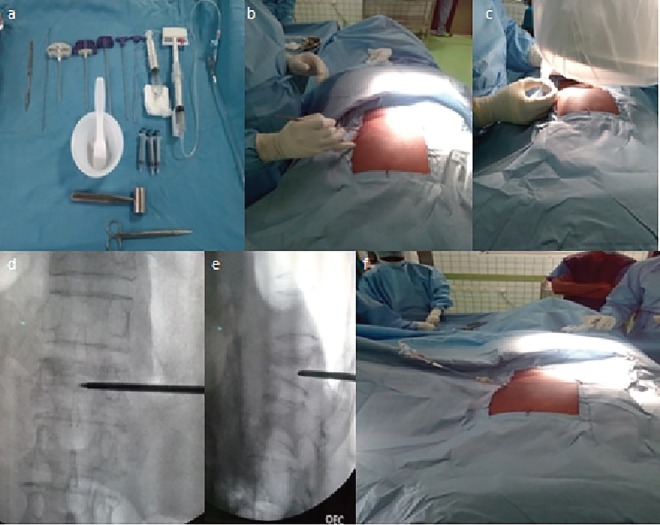
Operative technique for extrapedicular balloon kyphoplasty

Vertical lines were then drawn along the pedicle shadow. Pedicles were marked on the line A. Another point was marked on the line A at a distance x from midline, where x=distance of the centre of affected vertebral body from skin surface, measured on axial CT images (Figure 1C). This point acted as starting point and marked. Trocar and cannula were inserted from the starting point directed medially at an angle of 45° w.r.t. horizontal plane. Once the lateral wall of vertebra was reached, the needle was hammered under fluoroscopy guidance, so as to reach the center on both AP and lateral images (Figures 2D, and 2e). Trocar was then removed and guide wire was placed. Guide wire was then exchanged by a wider working cannula. Balloon tamp was then inserted through the cannula (Figure 2F) and positioned in the centere on AP and lateral images. 

**Fig. 3:**
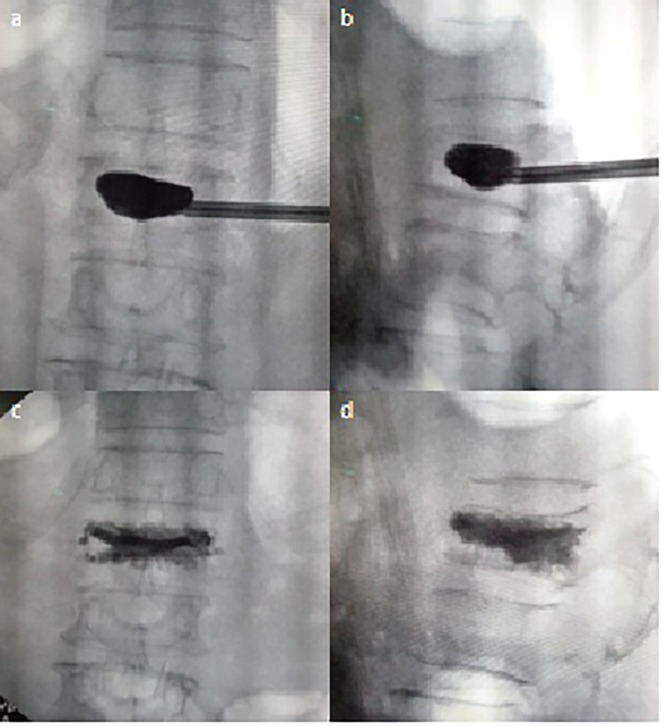
Balloon inflation/end plate elevation and cementation technique

Dye was injected to inflate the balloon to the point where maximum fracture reduction was achieved or balloon reaches cortical wall (Figures 3A and 3B). polymethyl methacrylate (PMMA) bone cement was then mixed. When cement reached the desired viscosity, the balloon was deflated and removed. Cement was injected slowly into the void, thus created using filler cannula under image intensifier guidance filler that was slowly pulled back as filling continued. Once filling was complete, filler was rotated approximately 360 degrees and taken out. Rotating the filler before pulling out reduced the chance of cement following the filler track (Figures 3C, and 3D). The procedure was aborted if there was evidence of any cement leak. Post-operative patients were allowed to walk with a supportive brace. Patients were discharged the next day with advises regarding treatment of osteoporosis.

## Results

**Fig. 4:**
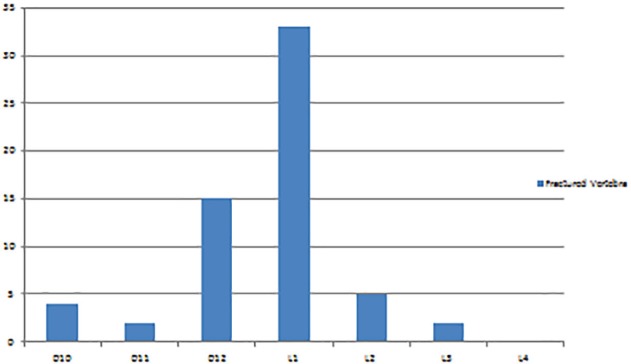
Frequency of vertebral involvement

Among 59 patients, 19 (32.20%) were male and 40 (67.80%) were female, with age range of 44 to 90 years (mean 62.29±10.06 years). They all satisfied our criteria undergoing balloon kyphoplasty. Age and sex distribution was shown in Figure 4. Totally, 28 (47.46%) patients were presented to outpatient department (OPD), and 31 (52.54%) patients were presented to the emergency room. Mean duration of presentation was 8.73 (range 2-12) days following injury. History of trivial injuries (like slip and fall) was present in 24 patients (40.68%) including fall from standing height in (18 patients, 30.51%), fall from bed (3 patients, 5.08%), sudden jerk while travelling (3 patients, 5.08%) and road traffic accidents (11 patients, 18.64%).

**Fig. 5:**
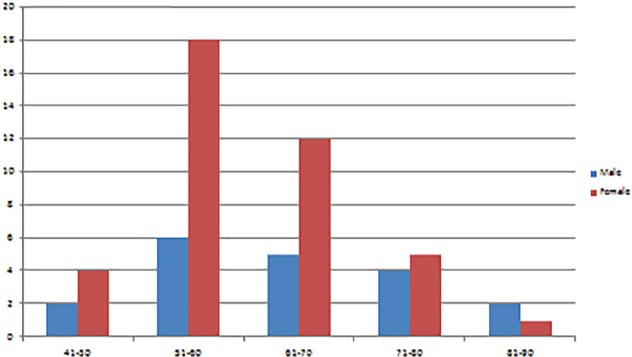
Demographic depiction of sex ratio and age distribution

As depicted in Figure 5, L1 was the most common vertebra involved (n=33, 54.10%), followed by D12 (n=15, 24.59%) vertebra. In our series, the mean segmental kyphosis correction following balloon kyphoplasty was 14.27° (SD=6.97) ranging from no change to a maximum of 30° reduction of kyphosis. Mean preoperatively segmental kyphosis was 22.81° (SD=10.93), and the mean post-balloon kyphoplasty segmental kyphosis was 8.54° (SD=11.51). At *p*<0.05, the result was significant with t=6.90498 and *p*<0.00001.

**Table1:** The pre-operative and post-operative values of anterior vertebral height, segmental kyphosis and VAS

	**Anterior vertebral height**	**Segmental kyphosis**	**VAS **
**Pre-op**	50.33%	22.81°	6.8
**Post-op**	79.61%	8.54°	2.4
	T=-12.08 *p*<0.00001	T=6.90 *p*<0.00001	*p*<0.05

**Table 2:** Comparison of demography, results and complications of our study with other studies

	**Demography**	**Anterior vertebral height restoration**	**Improvement in angle of kyphosis**	**VAS drop**	**Complications**
**Our series **	59 Patients, 19 males, 40 females	From 50.33% to 79.61%	From 22.81° to 8.54°=63% improvement	6.8 to 2.4=4.4	Asymptomatic cement leak in 15% cases
**Chia-Wei Yu** ***et al*. [**27**]**	187 patients 65 males, 122 females	From 52% to 74.5% *p*<0.05	From 14.4° to 6.7°=53% improvement	7.7 to 2.2=5.5	Cement leak in 11.5% cases
**Klezl ** ***et al*** **. [** 28 **]**	105 patients, 37 males, 68 females	NA	From 11.6° to 10.9°=6% improvement	8.2 to 4.4=3.8	11 patients ( 10.4%) with minor complications
**Saxena ** ***et al*** **. [** 29 **]**	135 patients	From 30.62% to 16. 19% (Loss of vertebral height)	17.41° to 10.59°=39% improvement	6.7 to 2.2=4.5	6 patients with cement embolism, 65 incidence of cement leak, 3 patients with adjacent level fracture
**Ledlie Jt ** ***et al*** **. [30]**	117 patients	Significant restoration; >10% increase in 84% of patients			Asymptomatic cement extravasation in 11.3% patients

Following osteoporotic compression fracture, anterior vertebral height decreased to an average of 50.33% (11.11-79.07%; sample SD=14.68). Postoperatively, anterior vertebral height improved to 79.61% of normal subjects (Range: 50-97.67%). At *p*<0.05, the result was significant with t=-12.08 and *p*<0.00001 (Table 1). In our series, the patients experienced quick and significant reduction in pain. Changes in VAS following treatment was significant with *p*<0.001, and the relief sustained during subsequent follow-up.

On first follow-up, >5° increase in Cobb’s angle was observed in 4 (6.78%) patients. At 1 year follow up, there were no significant (*p*>0.1) changes in radiological parameters compared to postoperative value. Anterior vertebral height and kyphotic angle were maintained to >95% of post-operative values. Overall, the incidence of cement leak in our study was 15.25% (9 patients), while 6 patients had asymptomatic paravertebral leak, and 2 patients had leak in disc space. One patient had venous embolism. There was no incidence of epidural leak. All patients had uneventful post-operation period. 

## Discussion

Fragility fracture of spine in osteoporotic patients is of great concern and accounts for 50% of the fractures. Intractable pain and deformity are the most common encountered problem and imparts the great repercussion over the quality of life [14, 15]. Conventional treatment of osteoporotic compression fracture (OCF) is by conservative mean, in the form of rest, pain killers, orthosis and rehabilitation. Galibert *et al*. introduced the PMMA injection (vertebroplasty) technique for first time to manage the cervical hemangioma [16]. 

In a subsequent year, this method was widely used by Jensen *et al*. for treating the osteoporotic compression fractures (OCF) [17]. Management of OCF is focused on pain control and functional recovery. Conservative treatment in the form of analgesia, pain, and bracing saves the patient from anesthesia and surgery related risks, but the drawbacks are complications related to immobilization, and kyphosis leading to sagittal imbalance, and an increased risk of OCF at other levels secondary to disturbed spinal column biomechanics [18-20]. 

Owing to above mentioned drawbacks cement augmentation of OCF in the form of vertebroplasty/balloon kyphoplasty is now an established form of treatment. Wang *et al*. concluded that high viscosity cement vertebroplasty and balloon kyphoplasty were safe and effective in improving quality of life and relieving pain [21]. Until recently, the biomechanical studies have been done mainly for the transpedicular approach for cement augmentation of OCF. Since now the extrapedicular approach has been developed recently; nowadays, the question becomes relevant to discuss the comparison of biomechanical studies of both. 

An *in vitro* cadaveric biomechanical study for transpedicular and extrapedicular approaches has shown that extrapedicular approach had superiority in height restoration [22]. The transpedicular approach posed difficulty in higher thoracic vertebral body (T9 and above) due to relatively smaller pedicle with and lateral angulation. So the above limiting factors are responsible for suboptimal balloon insertion by transpedicular approach due to more centric position of lateral cortex of pedicle. At these levels, the extrapedicular approach seems more relevant. Therefore, the extrapedicular approach is safer and more feasible even in higher thoracic regions [23]. 

Unilateral extrapedicular lumbar kyphoplasty has almost similar clinical outcomes for the thoracic as well as lumber vertebral compression fractures. But due to significant anatomical variations between these two vertebrae, a cautious unilateral extrapedicular approach is needed for lumbar region [24]. It is suggested to use extrapedicular approach for thoracic and transpedicular approach for lumbar vertebral compression fractures, because of potential risk of segmental artery damage in lumbar area by extrapedicular approach [25]. 

Cement leakage is a common problem, although it is mostly asymptomatic. Venous embolism and unrestricted posterior flow of cement in spinal canal can have serious outcomes too. Reported leakage rate is variable, ranging from 19% to 88% for vertebroplasty and 7% to 52% for balloon kyphoplasty. Studies using CT to investigate cement leakage usually report much higher rates. In our study, we observed 15.25% incidence of cement leakage with no leakage in epidural, foraminal or pedicular region. Vogl *et al*. has evaluated the role of CT guided kyphoplasty for reducing the cement leakage and found it an effective method [26].

Our series focused on clinical and radiological outcome analysis of OCF following balloon kyphoplasty through extrapedicular approach. Unilateral extrapedicular approach was found to be as effective as conventional transpedicular approach in restoring vertebral height, maintaining sagittal alignment and clinical recovery, the added advantages have less risk for epidural cement leak, the need for single balloon, less radiation exposure and a decreased duration of surgery. This approach is commonly utilized by interventional radiologists to perform vertebral biopsies.

Dorso-lumbar junction was the most common location (78.69%) in our series. Back pain, assessed by VAS score was shown to be improved significantly (*p*<0.05) within 24 hours of balloon kyphoplasty. Our results are similar to previously published literatures of balloon kyphoplasty as shown in Table 2.

Study of Chia-Wei Yu *et al*. had the less percentage of cement leakage in comparison to our study, but the VAS drop, anterior vertebral height restoration and improvement in angle of kyphosis were comparable [27]. Klezl Z *et al*. did not find any significant gain in kyphosis correction, other than VAS drop and minor complications [28]. Study of Saxena *et al*. had relatively higher complications and less kyphotic correction, but the VAS drop was similar to our study [29]. 

Study by Ledlie *et al*. also had comparable outcomes when compared with our series. So our series supported a significant improvement in VAS drop, sagittal alignment and anterior vertebral height restoration. We completed our study with few imitations. We had relatively small number of cases and lack of the comparison groups. Moreover, we did not include the upper thoracic level (T9 and above) and single level; therefore, we could not speculate the same result in upper thoracic level and multiple level OCF. Radiation exposure and duration of surgery were not calculated in our study too, and it seems to be another limitation of this study. 

Unilateral extrapedicular balloon kyphoplasty can be used as a relatively safer and practicable method for the management of osteoporotic vertebral compression fracture. Yet, we faced little difficulty in needle placement in kyphotic vertebrae. Furthermore, it is as effective as conventional techniques in restoring anterior vertebral height, alignment and angle of kyphosis along with added advantages of no risk of epidural cement leakage in cases with intact posterior cortex and need of single balloon. 

## Conflict of Interest:

None declared.
